# ATF3 Regulates Osteogenic Function by Mediating Osteoblast Ferroptosis in Type 2 Diabetic Osteoporosis

**DOI:** 10.1155/2022/9872243

**Published:** 2022-10-26

**Authors:** Yantao Zhao, Yunxia Du, Yijie Gao, Zhijie Xu, Dexiang Zhao, Maowei Yang

**Affiliations:** ^1^Department of Joint Surgery, Dalian Municipal Central Hospital, Dalian, Liaoning Province, China; ^2^China Medical University, Shenyang, Liaoning Province, China; ^3^Department of Rehabilitation Medicine, The Second Hospital of Dalian Medical University, Dalian, Liaoning Province, China; ^4^Dalian Medical University, Dalian, Liaoning Province, China; ^5^Department of Orthopedics, The First Hospital of China Medical University, Shenyang, Liaoning Province, China

## Abstract

**Purpose:**

Osteoporosis is a complication of type 2 diabetes, and it is characterized by reduced bone mass, augmented bone fragility, and increased risk of fracture, thus reducing patient quality of life, especially in the elderly. Ferroptosis has been implicated in the pathological process of type 2 diabetic osteoporosis (T2DOP), but the specific underlying mechanisms remain largely unknown. This study clarified the role of activating transcription factor 3 (ATF3) in T2DOP and explored its specific regulatory mechanism, providing a new treatment target for T2DOP.

**Methods:**

We cultured hFob1.19 cells in high glucose (HG, 35 mM) and knocked down ATF3 using short hairpin RNA (shRNA). We then measured cell viability, assessed morphology, quantified the expression of ATF3 and glutathione peroxidase 4 (GPX4), detected the levels of reactive oxygen species (ROS) and lipid peroxides, and determined the osteogenic function of osteoblasts. Cystine/glutamate antiporter (system Xc^−^) activity was evaluated by determining the expression of SLC7A11 and the levels of glutathione (GSH) and extracellular glutamate. We constructed a T2DOP rat model and observed the effect of ATF3 on ferroptosis and T2DOP by knocking down ATF3 using small interfering RNA (siRNA). Then, we evaluated the levels of iron metabolism, lipid peroxidation, and bone turnover in serum, detected the expression of ATF3, SLC7A11, and GPX4 in bone tissues, and assessed bone microstructure using microcomputed tomography.

**Results:**

ATF3 expression was increased in osteoblasts under HG condition and in T2DOP rats. Inhibiting the function of ATF3 increased GPX4 levels and reduced the accumulation of ROS and lipid peroxides. These changes inhibited the ferroptosis of osteoblasts and improved osteogenic function. In addition, HG induced ATF3 upregulation, resulting in decreased SLC7A11 expression and lower levels of intracellular GSH and extracellular glutamate.

**Conclusion:**

Osteoblast ferroptosis under HG conditions is induced by ATF3-mediated inhibition of system Xc^−^ activity, and these events contribute to T2DOP pathogenesis.

## 1. Introduction

Diabetes is a growing global public health concern, and type 2 diabetes accounts for approximately 90% of cases [1]. Epidemiological studies have shown that approximately 462 million individuals suffered from type 2 diabetes in 2017, corresponding to 6.28% of the world's population. With new cases diagnosed every year, the prevalence of type 2 diabetes is expected to increase to approximately 7% by 2030 [2]. Among the numerous complications of diabetes, osteoporosis can cause severe pain and deformity as well as impair mobility, physically and socioeconomically affecting patients [3, 4]. Therefore, it is necessary to further explore the pathogenesis and preventive measures of type 2 diabetic osteoporosis (T2DOP).

Ferroptosis, which was first proposed by Dixon et al. in 2012, is a form of iron-dependent cell death characterized by excessive accumulation of lipid peroxides and reactive oxygen species (ROS) [5]. Ferroptosis has been implicated in the pathophysiological processes of various conditions, such as neurodegenerative diseases, malignant tumors, ischemic diseases, and metabolic diseases [6–8]. Ferroptosis-inducing factors can reduce the activity of glutathione peroxidase 4 (GPX4) through various pathways, which leads to a serious decline in antioxidant capacity, ultimately causing oxidative cell death [9]. Because ROS accumulation can greatly affect the formation and survival of osteoblasts and inhibit their differentiation into bone cells, oxidative stress has been widely reported as an important factor contributing to T2DOP [10–12]. Wang et al. reported evidence of ferroptosis in the bone tissue of T2DOP rats, and treatment with ferroptosis inhibitors significantly decreases oxidative stress and improves osteoporosis symptoms [13]. While ferroptosis may be one of the main pathogenetic mechanisms of T2DOP, the specific pathways have not been fully elucidated.

As a member of the activating transcription factor (ATF)/cyclic adenosine monophosphate (cAMP) response element-binding (CREB) family, ATF3 contains a leucine zipper structure that can bind to the TGACGTCA consensus sequence of the cAMP response element (CRE) in many promoters. ATF3 expression remains at a low level under normal conditions, but its expression is rapidly induced following exposure to a variety of stimuli including oxidative stress, metabolic stress, and cell damage [14–16]. ATF3 is reportedly involved in the pathogenesis of diabetes and its complications through its transcription factor activity in response to oxidative stress. Jang et al. found that ATF3 inhibits the expression of pancreatic and duodenal homeobox 1 (PDX-1) in pancreatic *β* cells by binding to the ATF3 response element in its promoter, thereby inhibiting pancreatic *β* cell function [17]. These findings suggest that ATF3 is a negative regulator of PDX-1 expression and may play an important role in the occurrence and development of type 2 diabetes. Okamoto et al. reported increased ATF3 expression in the glomeruli and aortic endothelial cells of fatty diabetes model rats, and they proposed that the vascular complications of diabetes related to ROS may involve ATF-mediated pathological angiogenesis [18]. A recent study has demonstrated that ATF3, as one of the major endogenous solute carrier family 7 member 11 (SLC7A11) repressors, promotes erastin-induced ferroptosis by suppressing the cystine/glutamate antiporter (system Xc^−^) [19]. In the present study, we tested the hypothesis that ATF3 is involved in ferroptosis in osteoblasts under HG conditions and investigated the role of ATF3 in T2DOP to identify the specific regulatory mechanism.

## 2. Material and Methods

### 2.1. Cell Culture

The human osteoblast cell line hFob1.19 was obtained from the Cell Bank of the Chinese Academy of Sciences (Shanghai, China) and cultured in Dulbecco's modified Eagle medium and Ham's F12 medium (Gibco, Grand Island, NY, USA) containing 10% fetal bovine serum (Biological Industries, Kibbutz Beit-Haemek, Israel) at 37°C with 5% CO_2_ in a humidified incubator (Thermo Scientific, USA). The medium was replaced every other day. The hFob1.19 cells were dissociated by trypsin (Gibco, Grand Island, NY, USA) for subcultivation, and they were cultured at a cell density of 10^4^/cm^2^ for 24 h before their use in experiments. A subset of cells was grown in HG conditions (35 mM) for 48 h. To investigate the existence and mechanism of ferroptosis in osteoblasts, the ferroptosis inhibitor ferrostatin-1 (Fer-1) (5 *μ*M) (Sigma-Aldrich, Germany) and ferroptosis inducer erastin (5 *μ*M) (Cayman Chemical, Ann Arbor, Michigan) were added to the cell cultures.

### 2.2. Lentiviral Transfection

hFob1.19 cells were infected with NC-shRNA and ATF3-shRNA lentiviral particles (GeneChem Corporation, Shanghai, China). Briefly, 1 × 10^4^ cells were seeded in 6-well plates the day before infection. On the second day, 40 *μ*l of transfection reagent (Beyotime Biotechnology, Shanghai, China) and lentiviral particles (20 multiplicity of infection (MOI)) were added to the culture after the cells adhered to the plate. The infected cells were incubated overnight, and the medium was changed 24 h after infection. After 72 h, we observed the fluorescence intensity of cells with a fluorescence microscope to assess the transfection efficiency, and puromycin (2 *μ*g/ml) (Solarbio, Beijing, China) was added to screen stably transfected cells. The knockdown efficiency of ATF3 was determined by quantitative real-time polymerase chain reaction (qRT-PCR) and western blot analyses. The target sequences of ATF3-siRNA and NC-siRNA were 5′-UUGUGGACACUCACUAUUCTT-3′ and 5′-GUACCUUGACAGUACCGAUdTdT-3′, respectively.

### 2.3. RT-qPCR

We extracted total RNA with TRIzol reagent according to the manufacturer's instructions (Invitrogen, Carlsbad, CA, USA), and reverse transcription was performed using a PrimeScript RT kit containing gDNA Eraser (Takara, Shiga, Japan). Then, RT-qPCR was performed on an Applied Biosystems 7500 real-time PCR system (Applied Biosystems, Waltham, MA, USA) using a SYBR Premix Ex Taq II Kit (Takara) under the following conditions: 95°C for 30 s, followed by 45 cycles of 95°C for 15 s and 58°C for 34 s. Applied Biosystems 7500 software (version 2.3) was used for data analyses. We quantified the relative expression of target mRNA by comparing cycle threshold (Ct) values, and 18s was used as an internal control. The relative mRNA expression of target genes was calculated using the 2^-*ΔΔ*Ct^ method. The following primer sequences were used for qRT-PCR: ATF3 primer sequence, forward 5′-GTTGAGCTCGGGCTGGATAA-3′ and reverse 5′-CTATACTGCCGACCTGGCTG-3′; SLC7A11 primer sequence, forward 5′-GTGGTGTGTTTGCTGTC-3′ and reverse 5′-GCTGGTAGAGGAGTGTGC-3′; PTGS2 primer sequence, forward 5′-TGAGCATCTACGGTTTGCTG-3′ and reverse 5′-TGCTTGTCTGGAACAACTGC-3′; and 18S primer sequence, forward 5′-CCCGGGGAGGTAGTGACGAAAAAT-3′ and reverse 5′-CGCCCGCCCGCTCCCAAGAT-3′.

### 2.4. Cell Viability Analysis

Cell viability was determined by the Cell Counting Kit-8 (CCK-8, Beyotime Biotechnology) according to the manufacturer's instructions. hFob1.19 cells (100 *μ*l/well) were seeded into 96-well plates and incubated for 24 h at 37°C. Then, 10 *μ*l of CCK-8 regent was added to each well followed by incubation for 4 h, and the optical density (OD) values of cells in each well at 450 nm were measured using a microplate reader (Bio-Tek Instruments, Winooski, VT, USA). The relative cell viability was calculated using the following equation: relative cell viability = (test group OD value–blank group OD value)/(control group OD value–blank group OD value) × 100%.

### 2.5. Determination of Lactate Dehydrogenase (LDH) Activity

The LDH cytotoxicity assay kit (Beyotime Biotechnology) was used to determine the release of LDH to evaluate the amount of cell injury. After lysing cells, they were incubated in the dark with 60 *μ*l of LDH working solution at 25°C for 30 min. The OD values of the cells were measured at 490 nm using a microplate reader (Bio-Tek Instruments, Winooski, VT, USA).

### 2.6. Detection of ROS Levels

ROS assay kits (s0033s, Beyotime Biotechnology) were employed to detect intracellular ROS using the 2′,7′-dichlorodihydrofluorescein diacetate (DCFH-DA) fluorescent probe. First, hFob1.19 cells were cultured in different conditions for 24 h, and the probes were diluted 1 : 1000 in serum-free medium for cell resuspension. Cells were then incubated in a water bath at 37°C for 20 min. DCF fluorescence was detected by flow cytometry at an excitation wavelength of 488 nm and an emission wavelength of 525 nm. The cell fluorescence intensity represented the intracellular ROS level.

### 2.7. Lipid Peroxidation Assay

Lipid peroxidation levels were detected by malondialdehyde (MDA) assay kits (Beyotime Biotechnology, 532 nm), 4-HNE assay kits (ab238538, Abcam Inc., Cambridge, MA, USA, 450 nm), and a fluorescent lipid peroxidation probe (C11 BODIPY 581/591, Shanghai Mao Kang Biotechnology Co., Ltd., Shanghai, China) according to the manufacturer's instructions. The absorbance was measured by a microplate reader (Bio-Tek Instruments), and the fluorescence was determined using a fluorescence microscope (Olympus, Tokyo, Japan).

### 2.8. Measurement of Intracellular Iron Ions

To detect intracellular iron ions, cells were incubated with 5 *μ*M FeRhoNox-1 dye solution (Shanghai Mao Kang Biotechnology Co., Ltd., Shanghai, China) for 60 min followed by measurement of cellular fluorescence at an excitation of 532 nm and an emission at 570 nm. Fluorescence intensity was quantified by ImageJ software.

### 2.9. Alkaline Phosphatase (ALP) Detection

Floating cells were digested, collected by centrifugation, washed twice with PBS, and lysed using lysis buffer. The alkaline phosphatase (ALP) activity was measured using an ALP activity assay kit (Beyotime Biotechnology) according to the manufacturer's instructions. Absorbance was measured using a 96-well plate reader at a wavelength of 405 nm.

### 2.10. Determination of Reduced Glutathione (GSH) Levels

Commercial GSH/glutathione disulfide (GSSG) assay kits (Nanjing Jiancheng Bioengineering Institute, Nanjing, China) were purchased to detect intracellular GSH levels. Briefly, hFob1.19 cells were seeded at 2 × 10^5^ cells/well in 6-well plates and collected by centrifugation. Three times the volume of protein removal reagent solution was added to the cell precipitation, and cells were subjected to two freeze/thaw cycles using liquid nitrogen and a 37°C water bath. Cells were then placed at 4°C for 5 min and centrifuged at 10,000 × *g* for 10 min, and the supernatant was collected for determination of total GSH. GSH removal auxiliary liquid and GSH removal reagent working solution were added to the above supernatant to prepare GSSG measurement samples, and the absorbance of each well was measured using a microplate reader at a wavelength of 412 nm. A standard curve was generated based on the absorbance of the standard. The total GSH and GSSG concentrations were calculated in each group of samples, and the reduced GSH content was determined according to the formula.

### 2.11. Glutamate Release Assay

Amplex Red Glutamate Assay Kits (Thermo Fisher Scientific, USA) were used to measure the levels of extracellular glutamate. Briefly, 50 *μ*l of conditioned medium was added to a 96-well plate, and 50 *μ*l of working solution was added to each well. The plate was incubated at 37°C for 1 h in the dark, and the fluorescence was measured using a Cytation 3 imaging reader at an excitation wavelength of 530-560 nm and an emission wavelength of 590 nm. We first calculated glutamate release with reference to a glutamate standard curve, and the results were standardized based on the total cell number determined by CCK8 measurement at the end of the experiment.

### 2.12. Alizarin Red S Staining

The extracellular matrix calcium deposits were evaluated using Alizarin Red S staining for measurement of bone nodule formation after osteogenic differentiation. After 2 weeks of culture in osteogenic medium, hFob1.19 cells were routinely washed and fixed. Cells were stained with 40 mM Alizarin Red S solution (Sigma-Aldrich, Germany). Cells and nodule formation were imaged by phase-contrast microscopy (Nikon, Japan), and the density was assessed using Image-Pro Plus 6.0.

### 2.13. Western Blot Analysis

hFob1.19 cells were washed with PBS and lysed for 30 min in radioimmunoprecipitation assay buffer (Beyotime Biotechnology). Cells were centrifuged at 12,000 × *g* for 30 min at 4°C, and the supernatant was collected. The protein concentration was measured using the bicinchoninic acid method. Approximately 30 *μ*g of protein was separated by 10% sodium dodecyl sulfate-polyacrylamide gel electrophoresis and then transferred to nitrocellulose filter membranes (General Electric, Chicago, IL, USA). After blocking, the membranes were incubated with the appropriate primary antibodies at a dilution ratio of 1 : 1000 and overnight at 4°C. The membranes were then incubated with the corresponding secondary antibodies (at a dilution ratio of 1 : 4000) (Cell Signaling Technology, Danvers, USA) at room temperature for 2 h. The protein bands were detected using the EC3 Imaging System (UVP; Analytik Jena AG, Jena, Germany), and the relative intensity of protein bands was quantified using ImageJ software (National Institutes of Health, Bethesda, MD, USA). The following primary antibodies were used: ATF3 (ab254268), GPX4 (ab125066), osteocalcin (OCN) (ab133612), osteoprotegerin (OPG) (ab73400), SLC7A11 (ab175186), and *β*-actin (ab8226), which were all purchased from Abcam (Cambridge, UK).

### 2.14. Mitochondrial Membrane Potential Detection

A mitochondrial membrane potential detection kit (JC-1) (Biosharp, Hefei, China) was used to detect the mitochondrial membrane potential of each group of cells. After following the product instructions, cells were observed using a laser confocal microscope. The JC-1 monomer was detected at an excitation wavelength of 490 nm and an emission wavelength of 530 nm, while the JC-1 polymer was detected at an excitation wavelength of 525 nm and an emission wavelength of 590 nm. The relative ratio of red and green fluorescence was used to measure the ratio of mitochondrial depolarization.

### 2.15. Transmission Electron Microscopy (TEM)

Cells were collected, digested, centrifuged, and transferred to 6-well plates. After culture for 48 h in different conditions, cells were washed in cold PBS and fixed with 5% glutaraldehyde. Cells were then dehydrated and embedded, and 50 nm thick slices were prepared with an Ultracut S microtome (Leica Microsystems, Wetzlar, Germany). A transmission electron microscope (JEOL Co., Ltd., Tokyo, Japan) was utilized to observe the mitochondrial morphology in the sections.

### 2.16. Experimental Animals

Pathogen-free Sprague-Dawley (SD) rats were obtained from China Medical University, Department of Experimental Animals (Animal Certificate Number: SCXK (Liaoning) 2008-0005). The rats were fed with a high-fat diet for 2 months and then intraperitoneally injected with 30 mg/kg streptozotocin (S0130, Sigma-Aldrich, St. Louis, MO, USA) to establish a type 2 diabetes model. Successful establishment was confirmed when the fasting blood glucose (FBG) exceeded 7.8 mmol/l, and the insulin sensitivity index decreased. The rats were kept on a high-fat diet for 2 months to establish the T2DOP rat model, and at the beginning of the third month, the rats were injected with 200 *μ*l of ATF3-siRNA (Ruibo Biological Technology, Guangzhou, China) via tail vein injection every 2 days for 2 weeks to establish a model of ATF3 interference after T2DOP. After feeding for 2 weeks, the rats were sacrificed, and the femoral tissues were placed in ethylenediaminetetraacetic acid for decalcification, fixed in 4% formalin after 2-3 weeks, and then stored at 4°C. Control rats were supplied with normal food and water under normal laboratory conditions, and all rats were housed in a temperature-controlled room (22 ± 3°C) with a 12 h light/dark cycle. During this experiment, the weight of the rats was in the range of 220-270 g, and the blood glucose did not exceed the range of 5-18 mmol/l. After feeding for 3 months, the control group was injected with 200 *μ*l of NC-siRNA. All protocols were performed in accordance with animal ethics requirements.

### 2.17. Ethics Statement

Animal experiments were approved by the Institutional Ethics Review Board of Dalian Municipal Central Hospital Affiliated to Dalian Medical University, and all animal procedures were in agreement with ethical requirements.

### 2.18. Microcomputed Tomography (Micro-CT) Assessment

After sacrificing the rats by cervical dislocation, the right femur was removed and placed in a tube with a diameter of 10 mm perpendicular to the scanning axis, and it was imaged under the following scanning parameters: 1024 × 1024 image matrix, 80 kV voltage, 80 *μ*A current, and 2.96 s exposure time. We selected a cancellous bone area (1.0 mm × 3.0 mm thick) from the distal growth plate, and we used the lowest threshold value extracted from 190 images to generate a reconstruction line. The images were then reconstructed using micro-CT, and the following parameters were determined: bone mineral density (BMD), trabecular number (Tb.N), trabecular thickness (Tb.Th), and trabecular bone volume per tissue volume (BV/TV).

### 2.19. Immunohistochemistry (IHC)

The bone tissue sections were deparaffinized in xylene and rehydrated in a graded series of ethanol. After incubating in 3% H_2_O_2_ at room temperature for 10 min followed by antigen recovery, the sections were blocked with 10% goat serum. The sections were then incubated with primary rabbit monoclonal anti-ATF3 (1 : 200; ab254268, Abcam, Cambridge, UK), anti-GPX4 (1 : 200; ab125066, Abcam), or anti-SLC7A11 (1 : 200; ab175186, Abcam) at 4°C overnight. The next day, the sections were incubated with secondary antibodies (SAP-9100, ZsBio, Beijing, China) for 2 h followed by an incubation with 0.1% DAPI for 5 min. After washing with PBS, images were acquired using a microscope (Leica Microsystems), and ImageJ software was used for semiquantitative analysis.

### 2.20. Determination of Serum Iron Ions, MDA, and GSH

The corresponding kits were purchased from Nanjing Jiancheng Bioengineering Institute. Serum samples were collected to measure the levels of iron ions, MDA, and GSH in rats. According to the instructions, the OD values were measured at 520 nm, 532 nm, and 412 nm, respectively.

### 2.21. Detection of Bone Metabolism Markers

The serum bone metabolism markers alkaline phosphatase (ALP), procollagen type I N propeptide (PINP), and *β*-isomerized type I collagen C-telopeptide (*β*-CTx) were detected using the corresponding enzyme-linked immunosorbent assay (ELISA) kits (Fankew, China). The standards and samples were added to the enzyme-labeled coated plate and reacted for 30 min at 37°C. After washing the plate five times, the enzyme-labeled reagent was added and reacted for 30 min at 37°C. Color-developing solutions A and B were added to the plate for 10 min at 37°C. Finally, stop solution was used to stop the reaction, and the OD values were detected at 450 nm using a microplate reader (Bio-Tek Instrument, Winooski, VT, USA). We calculated the concentration of samples by drawing a curve based on the concentration of the standard substance and the corresponding OD value.

### 2.22. Statistical Analysis

Each experiment was repeated at least three times, and all data obtained from the experiment were expressed as mean ± standard deviation (SD). GraphPad Prism 7 software (GraphPad Software, Inc., San Diego, CA, USA) was used for statistical analysis and graphing. Differences between two groups were compared with Student's *t*-tests, and analysis of variance was used for the comparisons among multiple groups. *P* < 0.05 was considered statistically significant.

## 3. Results

### 3.1. Ferroptosis Was Induced by HG in hFob1.19 Cells

**Figure 1:**
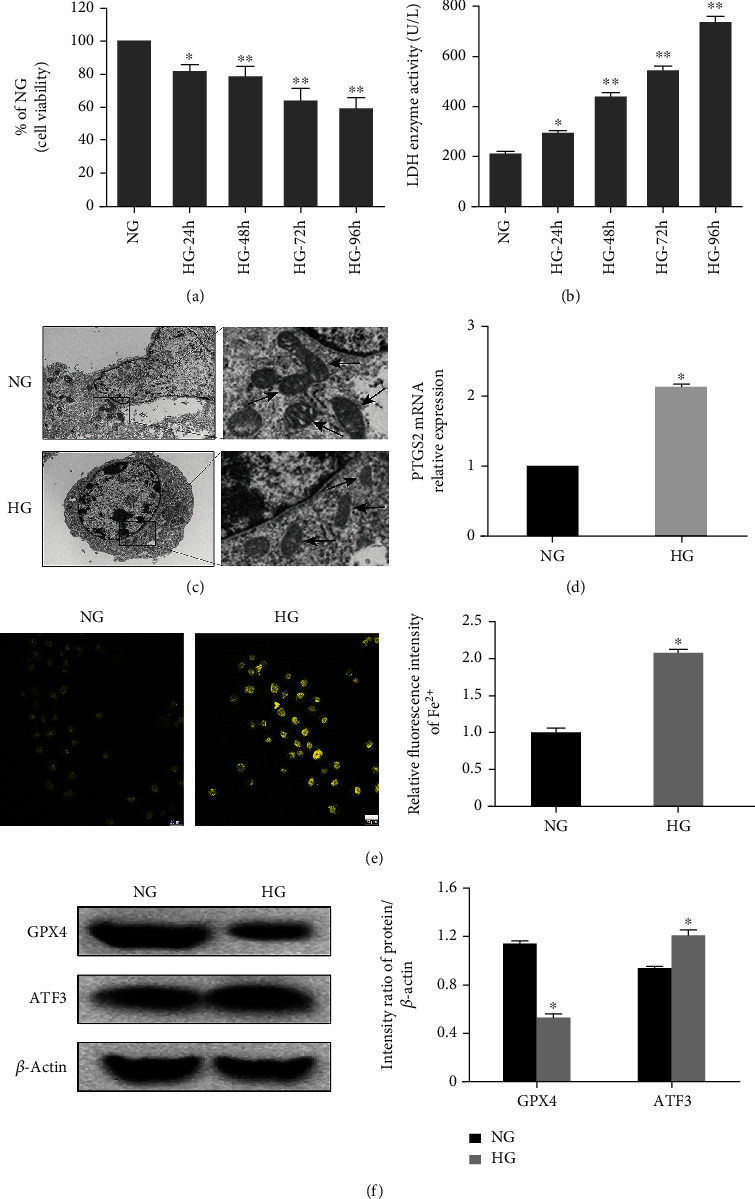
Ferroptosis is induced by HG in hFob1.19 cells. (a) Cells were treated with HG for 24 h, 48 h, 72 h, and 96 h. Cell viability was measured using the CCK-8 assay, and the data were expressed as values relative to the NG group. (b) The LDH enzyme activity was quantified using the LDH content kit. (c) Transmission electron microscopy images of hFob1.19 cells treated with HG and NG. Ferroptosis was only observed in the HG group. (d) HG caused an increased level of PTGS2 mRNA. (e) Intracellular iron ions were detected using FeRhoNox-1 fluorescent probe. Scale bar = 20 *μ*m. (f) hFob1.19 cells were treated with NG and HG, respectively. The protein levels of ferroptosis-related protein GPX4 and ATF3 were determined by western blot. All data are presented as the mean ± SD of three independent experiments. ^∗^*P* < 0.05 vs. NG and ^∗∗^*P* < 0.01 vs. NG.

We treated hFob1.19 cells with HG (35 mM) and normal glucose (NG, 17.5 mM) [20], and we determined the optimal time (48 h) of HG treatment using the CCK-8 cell viability assay (Figure 1(a)) and LDH release assay (Figure 1(b)). After 48 h of HG treatment, the mitochondrial morphology in osteoblasts, as detected by TEM, was consistent with the induction of ferroptosis (Figure 1(c)). Moreover, we found increased levels of PTGS2 mRNA and intracellular iron ions (Figures 1(d) and 1(e)), as well as decreased expression of GPX4 in the HG group (Figure 1(f)), which were the markers of ferroptosis, suggesting that HG may induced ferroptosis in osteoblasts.

**Figure 2:**
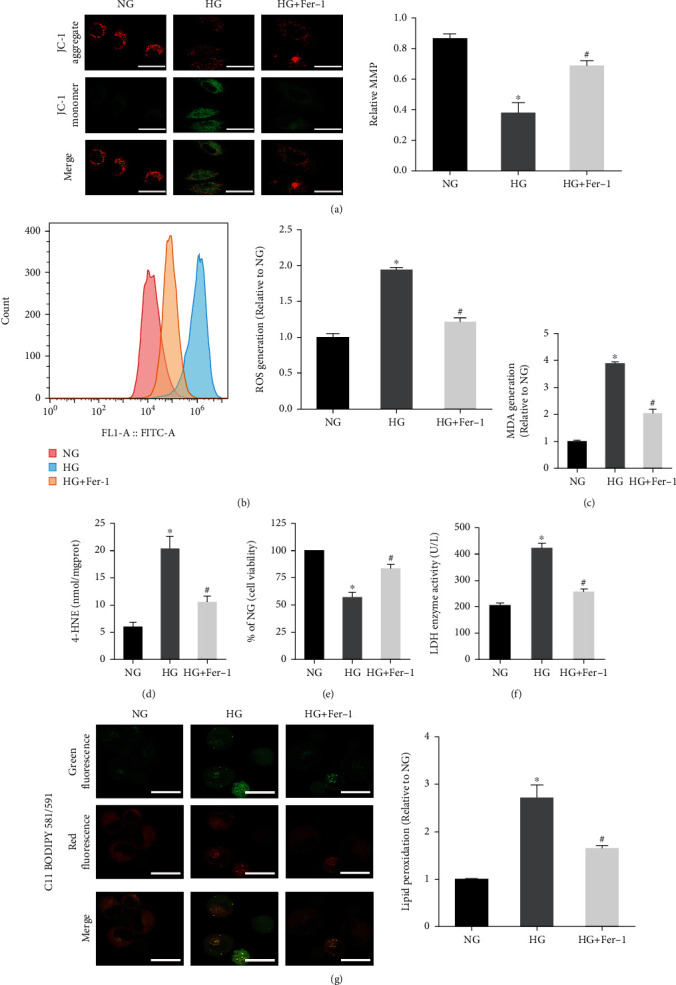
Effect of Fer-1 on osteoblasts treated with HG. (a) JC-1 staining showed a decreased level of the mitochondrial membrane potential in osteoblasts under HG conditions. Scale bar = 20 *μ*m. (b) Flow cytometry analysis of intracellular ROS generation. (c) Lipid peroxidation was detected using the MDA assay kits. (d) Lipid peroxidation was detected by the 4-HNE assay kits. (e) Fer-1 reversed the decrease of osteoblast viability induced by HG, measured by CCK-8 assay. (f) The amount of cell injury was evaluated by LDH release assay. (g) Detection of lipid peroxidation by C11 BODIPY 581/591 fluorescent probe. Scale bar = 20 *μ*m. All data are presented as the mean ± SD of three independent experiments. ^∗^*P* < 0.05 vs. NG and ^#^*P* < 0.05 vs. HG.

Ferroptosis is an iron-dependent form of cell death characterized by lipid peroxidation. After HG treatment, we found a lower mitochondrial membrane potential (Figure 2(a)) and an increased ROS level in hFob1.19 cells (Figure 2(b)). At the same time, the lipid peroxidation level was measured using MDA kits (Figure 2(c)), 4-HNE kits (Figure 2(d)), and fluorescent lipid peroxidation probe (Figure 2(g)), and we found the accumulation of lipid peroxides in the HG group cells. To further verify that the decrease of cell viability induced by HG was due to ferroptosis, the HG group cells were treated with ferroptosis inhibitor (Fer-1). We found that the addition of Fer-1 to HG-treated hFob1.19 cells remarkably increased mitochondrial membrane potential (Figure 2(a)) and decreased the production of ROS (Figure 2(b)) and lipid peroxides (Figures 2(c), 2(d), and 2(g)), then rescuing osteoblasts from death induced by HG (Figures 2(e) and 2(f)). To sum up, we concluded that ferroptosis was involved in the process of HG-induced cell death, providing a new treatment option for the clinical treatment of T2DOP.

### 3.2. HG Decreased Osteogenic Function by Inducing Ferroptosis

**Figure 3:**
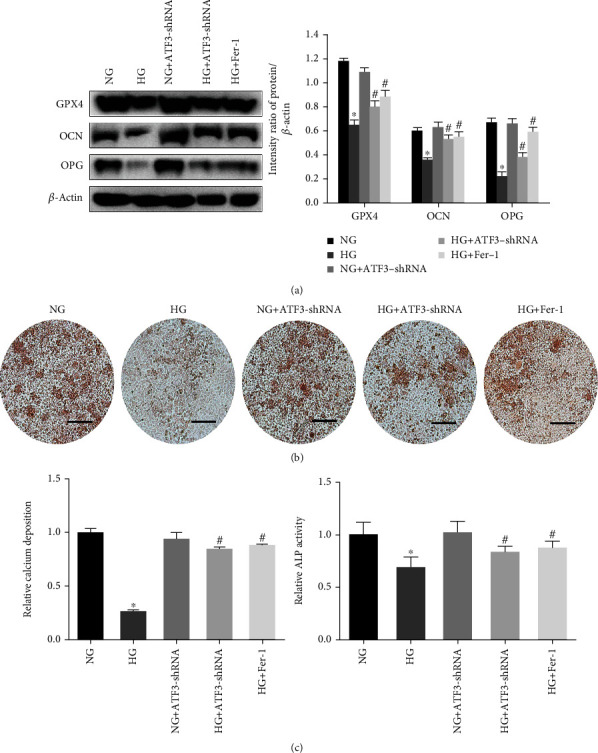
HG decreased the osteogenic function of hFob1.19 cells. (a) Western blots of GPX4, OCN, and OPG expression levels. (b) Mineralized extracellular matrix in hFob1.19 cells after osteogenic differentiation for 2 weeks under different conditions, shown by Alizarin Red S staining. Scale bar = 200 *μ*m. (c) ALP activity was determined after indicated treatments. All data are presented as the mean ± SD of three independent experiments. ^∗^*P* < 0.05 vs. NG and ^#^*P* < 0.05 vs. HG.

To assess the osteogenic function of osteoblasts, we detected several osteogenesis-related indicators. Compared to the NG group, western blot analysis showed that the OCN and OPG expression levels were downregulated in the HG group (Figure 3(a)), and ALP detection showed a decreased activity of ALP in the HG group (Figure 3(c)). After 2 weeks of culture, cells treated with HG showed decreased mineralized nodule formation compared to the NG group (Figure 3(b)).

However, treatment of the cells with Fer-1, which inhibits ferroptosis, blocked the decrease of osteogenic function induced by HG. These results suggested that the decrease of osteogenic function of osteoblasts induced by HG may be related to ferroptosis.

### 3.3. Effects of ATF3 on Ferroptosis and Osteogenic Function under HG Condition

**Figure 4:**
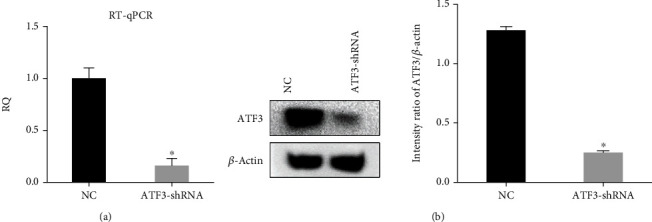
Verification of ATF3 knockdown efficiency. (a) ATF3 knockdown efficiency was verified by RT-qPCR. (b) ATF3 knockdown efficiency was verified by western blot analysis. All data are presented as the mean ± SD of three independent experiments. ^∗^*P* < 0.05 vs. NC.

After treatment with HG for 48 h, western blot analysis revealed an increased level of ATF3 protein in osteoblasts compared to the NG group (Figure 1(f)). We observed the effect of ATF3 on osteoblasts by lentiviral transfection using ATF3-shRNA to downregulate the expression of ATF3, which was confirmed by RT-qPCR and western blot analysis (Figures 4(a) and 4(b)).

**Figure 5:**
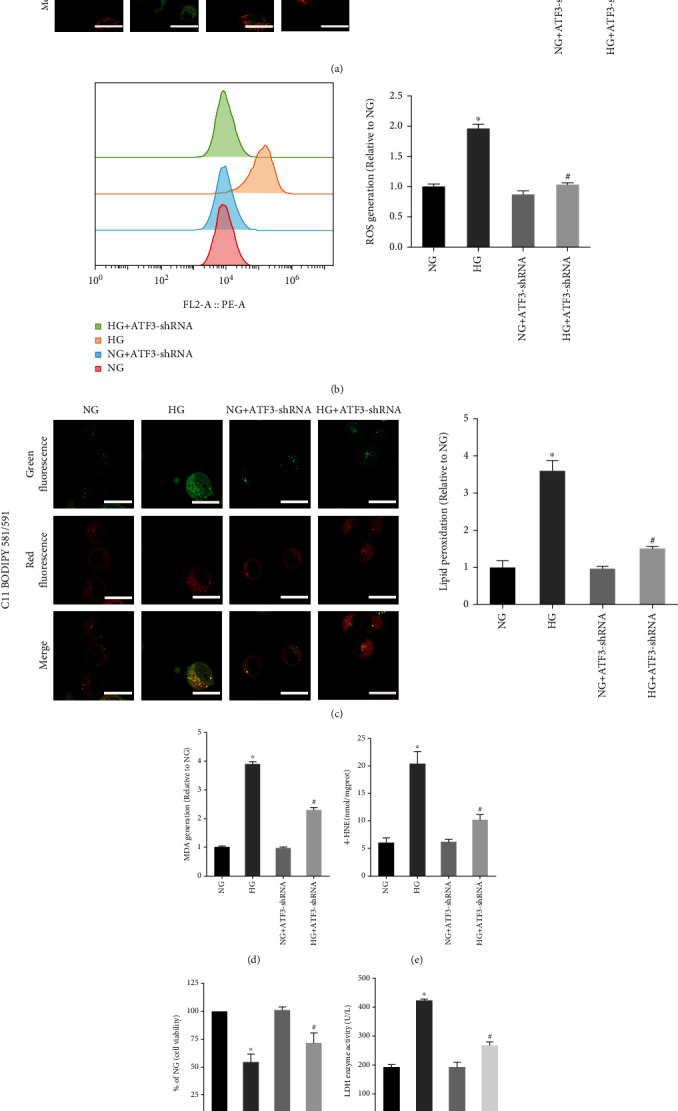
Effects of ATF3 on ferroptosis of osteoblasts under HG condition. (a) Mitochondrial membrane potential was determined by JC-1 staining. Scale bar = 20 *μ*m. (b) Intracellular ROS generation detected using fluorescent probe (DCFH-DA). (c) Lipid peroxidation was detected by C11 BODIPY 581/591 fluorescent probe. Scale bar = 20 *μ*m. (d) Lipid peroxidation was determined by measuring MDA levels. (e) Lipid peroxidation was determined by measuring 4-HNE levels. (f) Cell viability was determined by CCK-8 assay. (g) The LDH enzyme activity was determined using the LDH content kit. All data are presented as the mean ± SD of three independent experiments. ^∗^*P* < 0.05 vs. NG and ^#^*P* < 0.05 vs. HG.

The results showed that knockdown of ATF3 reversed the changes of osteoblasts induced by HG (mitochondrial membrane potential decreased, GPX4 expression decreased, and ROS and lipid peroxide levels increased) (Figures 3(a) and 5(a)–5(e)), indicating that ATF3 knockdown attenuated ferroptosis. Furthermore, knockdown of ATF3 in the HG group resulted in increased OCN and OPG expression (Figure 3(a)), increased ALP activity (Figure 3(c)), increased mineralized nodule formation (Figure 3(b)), increased cell viability (Figure 5(f)), and decreased LDH enzyme activity (Figure 5(g)). These results suggested that downregulated ATF3 reduced lipid peroxidation and inhibited ferroptosis, thereby improving the osteogenic function of osteoblasts under HG condition.

### 3.4. ATF3 Mediated HG-Induced Ferroptosis by Inhibiting the Function of System Xc^−^

System Xc^−^ is an amino acid antiporter that mediates the exchange of extracellular cystine and intracellular glutamate on the cell membrane, and it is composed of two subunits of the xCT light chain (catalytic subunit, encoded by the SLC7A11 gene) and the 4F2hc heavy chain (chaperone subunit, encoded by the SLC3A2 gene) [21, 22]. Since the light chain encoded by SLC7A11 is responsible for the primary transport activity and the heavy chain subunit SLC3A2 mainly functions as a chaperone protein, the expression level of SLC7A11 is generally positively correlated with the activity of the antiporter, playing a key role in preventing ferroptosis induced by lipid peroxidation.

**Figure 6:**
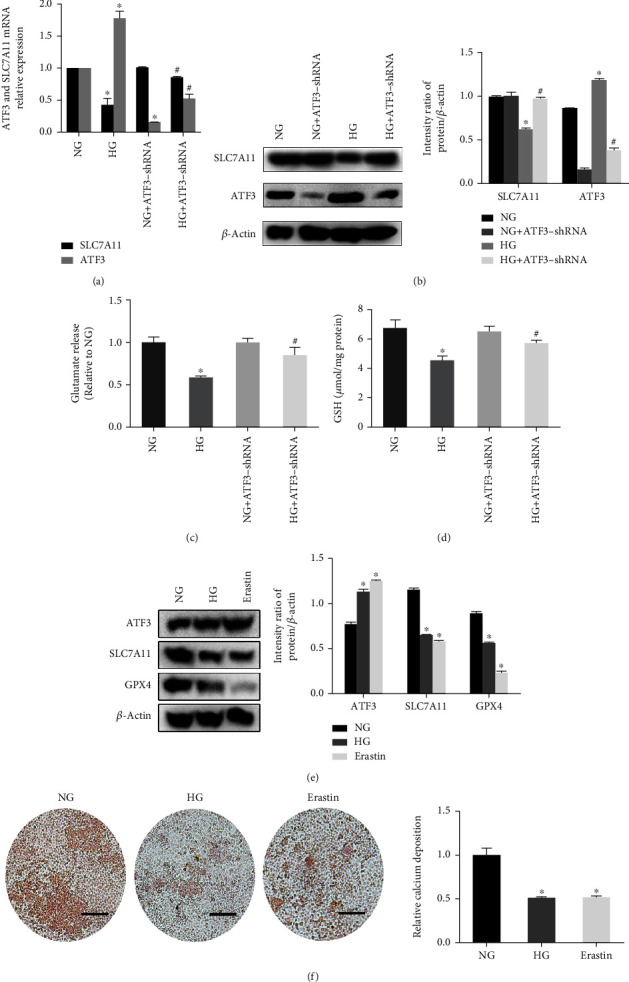
ATF3 mediated HG-induced ferroptosis by inhibiting system Xc^−^ function. (a) Relative ATF3 mRNA and SLC7A11 mRNA levels were assayed using RT-qPCR. (b) Western blot showed increased level of ATF3 protein and decreased level of SLC7A11 protein under HG condition. (c) Relative level of glutamate release was assayed. (d) Assessment of GSH levels in different conditions. (e) Expression of ATF3 protein and SLC7A11 protein measured by western blot in NG, HG, and erastin group. (f) Alizarin Red S staining in NG, HG, and erastin group. Scale bar = 200 *μ*m. All data are presented as the mean ± SD of three independent experiments. ^∗^*P* < 0.05 vs. NG and ^#^*P* < 0.05 vs. HG.

RT-qPCR analysis revealed an increased mRNA level of ATF3 and a decreased mRNA level of SLC7A11 after HG exposure, and knockdown of ATF3 reversed these effects (Figure 6(a)). At the same time, western blot analysis demonstrated that HG decreased the expression of SLC7A11 protein (Figure 6(b)). After 48 h of HG treatment, glutamate release assays showed that the extracellular glutamate level and intracellular GSH level were significantly decreased (Figures 6(c) and 6(d)). In contrast, knockdown of ATF3 resulted in upregulation of SLC7A11 protein expression (Figure 6(b)) and increased extracellular glutamate and GSH (Figures 6(c) and 6(d)). Therefore, we concluded that HG decreased system Xc^−^ activity by inhibiting SLC7A11 expression, thereby restricting glutamate-cystine transport and inhibiting the synthesis of intracellular GSH.

Studies have shown that erastin can induce ferroptosis by inhibiting the function of system Xc^−^ [19]. In our research, we treated NG group cells with erastin and found that erastin increased ATF3 expression, decreased GPX4 expression, decreased SLC7A11 expression (Figure 6(e)), and reduced mineralized nodule formation (Figure 6(f)), which were consistent with the results caused by HG. These findings indicated that the upregulation of ATF3 and osteogenic dysfunction induced by HG was similar to the ferroptosis inducer erastin.

### 3.5. Establishment of the T2DOP Rat Model

**Figure 7:**
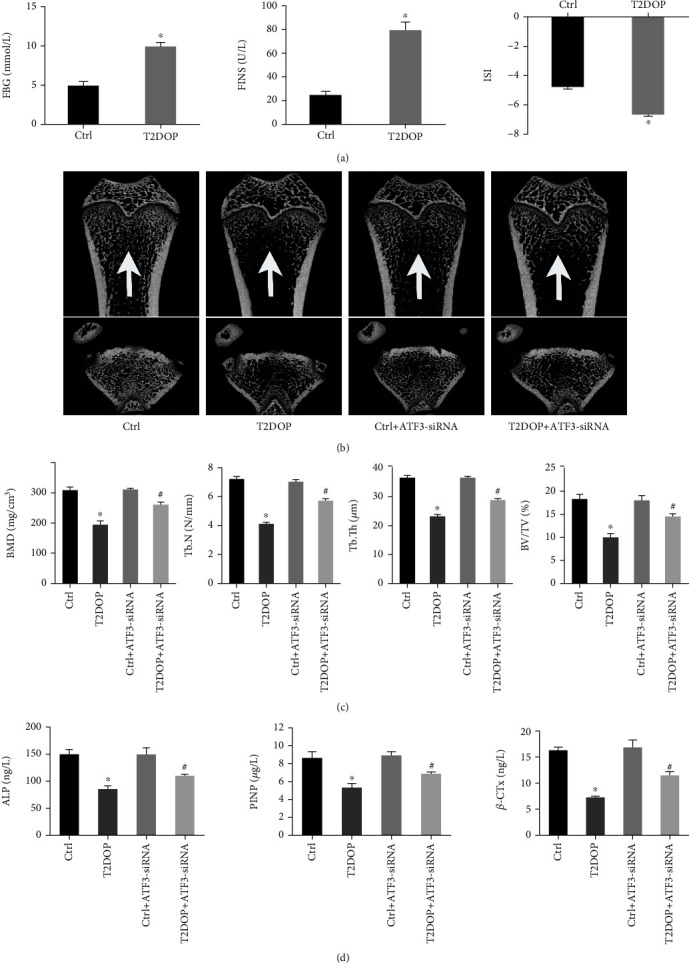
Establishment of the T2DOP rat model. Sixty SD rats were divided into four groups (*n* = 15). (a) FBG and FINS increased and ISI decreased in T2DOP rats. (b) Micro-CT analysis of the distal metaphyseal femur region. (c) Micro-CT-based quantification of BMD, Tb.N, Tb.Th, and BV/TV. (d) Serum levels of the bone metabolism markers ALP, P1NP, and *β*-CTx were determined by ELISA. ^∗^*P* < 0.05 vs. Ctrl and ^#^*P* < 0.05 vs. T2DOP.

The T2DOP rat model was induced through high-fat feeding combined with intraperitoneal injection of streptozotocin [23]. We evaluated FBG, fasting insulin (FINS), and the insulin sensitivity index (ISI) to confirm that the model was successfully established (Figure 7(a)). Then, we assessed bone microstructure indicators using micro-CT and detected serum levels of bone metabolism markers, including ALP, P1NP, and *β*-CTx by ELISA. Compared to the control rats, the results showed that BMD, BV/TV, Tb.N, and Tb.Th were significantly reduced in experimental group rats (Figures 7(b) and 7(c)), and the serum levels of ALP, P1NP, and *β*-CTx were significantly lower in experimental group rats (Figure 7(d)). These findings suggested that both bone quality and bone turnover were significantly worse in the rat model of T2DOP.

### 3.6. Ferroptosis and Increased ATF3 Expression in T2DOP Rats

**Figure 8:**
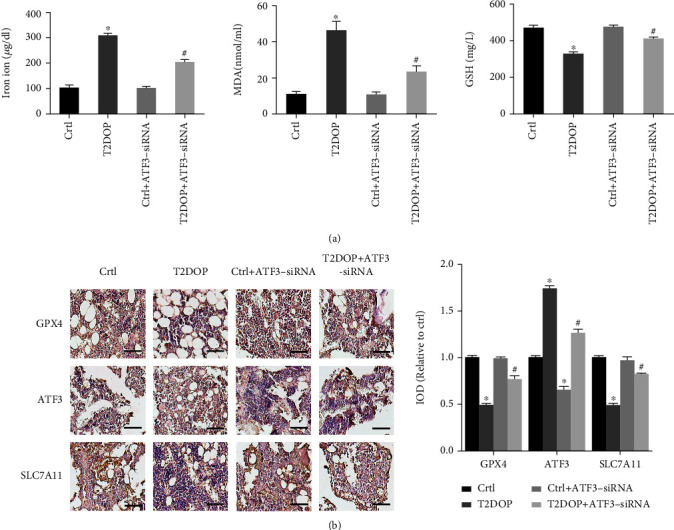
Effect and potential mechanism of ATF3 on T2DOP. Sixty SD rats were divided into four groups (*n* = 15). (a) Determination of iron ion, MDA, and GSH in serum. (b) IHC for GPX4, ATF3, and SLC7A11 in four groups of rats. Scale bar = 100 *μ*m. ^∗^*P* < 0.05 vs. Ctrl and ^#^*P* < 0.05 vs. T2DOP.

In T2DOP rats, the serum contents of iron ions and MDA increased, while GSH level decreased (Figure 8(a)). IHC analysis showed decreased expression of GPX4 and SLC7A11 but increased expression of ATF3 in the bone tissue of T2DOP rats (Figure 8(b)), indicating that ferroptosis was involved in the pathogenesis of T2DOP. Thus, inhibition of ferroptosis may significantly improve T2DOP [13, 24].

We next knocked down ATF3 in T2DOP rats using ATF3-siRNA. In ATF3-deficient T2DOP rats, the serum contents of iron ions and MDA were decreased, and the serum contents of GSH were increased (Figure 8(a)). The data of IHC showed that the GPX4 and SLC7A11 expression levels were significantly increased compared to T2DOP rats, suggesting an improvement in ferroptosis and system Xc^−^ activity (Figure 8(b)). To analyze the effects of ATF3 on osteoporosis, we assessed the bone formation markers. ATF3 knockdown resulted in increased values of BMD, Tb.N, Tb.Th, and BV/TV as well as elevated serum levels of ALP, P1NP, and *β*-CTx, which indicated improved bone quality and bone turnover (Figures 7(b) and 7(c)). These results demonstrated that inhibiting the function of ATF3 can effectively improve ferroptosis and reduce the severity of T2DOP.

## 4. Discussion

Among the many complications of type 2 diabetes, osteoporosis has received increasing attention because it increases vulnerability to fragility fractures and has high disability and mortality rates [25, 26]. Although the role of BMD in T2DOP has been controversial, the risk of osteoporotic fractures in patients with type 2 diabetes is still significantly higher than that of the general population due to decreased bone quality and increased bone fragility [27, 28]. Therefore, it is of great significance to elucidate the pathogenesis of T2DOP.

Studies have shown that oxidative stress affects bone homeostasis, ultimately leading to the development of osteoporosis [29, 30]. Jeney reported that iron overload significantly influences both increased bone resorption and decreased bone formation [31]. Because iron overload and accumulation of lipid peroxides are considered to be important characteristics of ferroptosis, they may play a significant role in the pathogenesis of T2DOP. Ma et al. found that HG causes lipid peroxide accumulation and induces ferroptosis in osteoblasts, and they demonstrated that treatment with ferroptosis inhibitors significantly improves T2DOP [24]. Similarly, the present study showed that HG induced ferroptosis in osteoblasts, leading to reduced cell viability and osteogenic function.

As a glutathione peroxidase, GPX4 catalyzes the combination of lipid hydroperoxide with the sulfhydryl group of reduced glutathione, which converts harmful substances into nontoxic lipid alcohols, thereby preventing ROS chain reaction. Thus, GPX4 is considered as a determinant mediator of ferroptosis. Numerous studies have confirmed that degradation and inactivation of GPX4 prevent lipid oxides from being metabolized by the GSH reductase reaction catalyzed by GPX4, resulting in lipid peroxide accumulation [32–34]. Some compounds, such as RSL3, ML162, erastin, and FINO2, have been reported to be inhibitors of GPX4 and sufficiently induce ferroptosis [35, 36]. A recent study by Wang et al. found that HG induces ferroptosis in osteoblasts by inhibiting GPX4 activity, which was consistent with our results [13]. Therefore, inhibiting GPX4 inactivation is critical for preventing ferroptosis.

The system Xc^−^-GSH-GPX4 axis is recognized as a critical part of lipid peroxide elimination. The present study showed that HG led to decreased levels of intracellular GSH and extracellular glutamate, resulting in inhibition of GPX4 expression. Therefore, we speculated that system Xc^−^, which is upstream of GSH synthesis, may be the mechanism by which HG induces ferroptosis. We then measured SLC7A11 mRNA and protein expression, and we confirmed that both decreased under HG, suggesting that HG induces ferroptosis in osteoblasts by inhibiting system Xc^−^ function.

SLC7A11 is regulated by a variety of transcription factors, including TP53, Nrf2, BACH1, and ATF4 [37–40]. Wang et al. showed that ATF3 is one of the main inhibitors of SLC7A11, which dampens system Xc^−^ activity through binding to and repressing the SLC7A11 promoter, thereby promoting erastin-induced ferroptosis of retinal pigment epithelial cells [19]. At the same time, ATF3 has been reported to be involved in brucine-induced ferroptosis of glioma cells [41]. Wang et al. found that ATF3 was increased in acute kidney injury, and ATF3 knockout significantly increased SLC7A11 and GPX4 levels, thereby increasing the viability of proximal tubule epithelial cells in the kidney [42]. The present study suggested that ATF3 may be involved in T2DOP by mediating ferroptosis in osteoblasts, revealing a new role of ATF3.

The present study demonstrated ferroptosis and increased ATF3 expression in osteoblasts exposed to HG and in T2DOP rats. Notably, lipid peroxide accumulation, decreased GPX4 expression, and weakened osteogenic function were significantly improved after knocking down ATF3. We further investigated the relationship between ATF3 and SLC7A11, and we found that the HG-induced increase of ATF3 significantly inhibited SLC7A11 expression and hindered glutamate/cystine transport. Therefore, we conclude that ATF3 promotes HG-induced ferroptosis of osteoblasts by inhibiting system Xc^−^. However, the regulatory mechanism of ATF3 and whether there is an association with other SLC7A11-related transcription factors remain to be explored.

## 5. Conclusion

In summary, the present study suggested that HG induces ferroptosis of osteoblasts through ATF3-mediated inhibition of glutamate/cystine exchange activity by system Xc^−^. These results indicated that ATF3 may be a key regulator of osteoblastic ferroptosis in the T2DOP rat model, providing a new potential target for the treatment and prevention of T2DOP.

## Data Availability

All original data are available from the corresponding author.
